# High Tech, High Risk: Tech Ethics Lessons for the COVID-19 Pandemic Response

**DOI:** 10.1016/j.patter.2020.100102

**Published:** 2020-10-09

**Authors:** Emanuel Moss, Jacob Metcalf

**Affiliations:** 1 Data & Society Research Institute, New York, NY 10010, USA; 2 CUNY Graduate Center, New York, NY 10016, USA

## Abstract

The COVID-19 pandemic has, in a matter of a few short months, drastically reshaped society around the world. Because of the growing perception of machine learning as a technology capable of addressing large problems at scale, machine learning applications have been seen as desirable interventions in mitigating the risks of the pandemic disease. However, machine learning, like many tools of technocratic governance, is deeply implicated in the social production and distribution of risk and the role of machine learning in the production of risk must be considered as engineers and other technologists develop tools for the current crisis. This paper describes the coupling of machine learning and the social production of risk, generally, and in pandemic responses specifically. It goes on to describe the role of risk management in the effort to institutionalize ethics in the technology industry and how such efforts can benefit from a deeper understanding of the social production of risk through machine learning.

## The Bigger Picture

This paper describes the coupling of machine learning and the social production of risk in general, with specific illustrations drawn from machine learning applications in response to the COVID-19 pandemic. As the COVID-19 pandemic has drastically reshaped society around the world, many have looked to machine learning as a technology capable of addressing large problems at scale, and machine learning applications have been seen as desirable interventions in mitigating the risks of the pandemic disease. However, machine learning, like many tools of technocratic governance, is deeply implicated in the social production and distribution of risk. Therefore, the role of machine learning in the production of risk must be considered as engineers and other technologists develop tools for the current crisis. The paper concludes by describing the role of risk management in the effort to institutionalize ethics in the technology industry, and how such efforts can benefit from understanding the social production of risk through machine learning.

## Teaser

This paper describes the coupling of machine learning and the social production of risk, with specific illustrations drawn from machine learning applications in response to the COVID-19 pandemic. It goes on to describe the role of risk management in the effort to institutionalize ethics in the technology industry, and how such efforts can benefit from a deeper understanding of the social production of risk through machine learning.

## Machine Risk Society

Ulrich Beck begins his 1992 book *Risk Society* by saying that “the social production of wealth is accompanied by the social production of risks.”^1^ He describes how economic and social inequality overlaps with vulnerability to the risks produced by the scientific and technological practices that underlie the wealth of the modern world. The COVID-19 pandemic illustrates Beck's thesis in excruciating detail, as do the applications of machine learning that attempt to address the many concerns of the pandemic. Indeed, machine learning has become a key technological practice that produces, and redistributes, risk across society.

The risk of pandemic disease is socially produced—the SARS-CoV-2 virus traveled on jet planes and cruise ships through global networks of trade and tourism,^2^ but the shareholders of transportation companies have been insulated from the risk to their business through enormous government cash bailouts. Populations that may be most vulnerable to disease have the fewest medical resources,^3^ and countries with capacity to manufacture any coronavirus vaccine are less likely to be able to afford it for their own citizens.^4^ In the US, many of those most likely to come into contact with others during lockdown—the essential grocery clerks, home health workers, warehouse workers, and delivery workers—are the least likely to have sick leave and work-from-home policies.^5^ Meanwhile, those who can afford to use delivery services while they work from home are able to enjoy greatly reduced risks of contracting COVID-19, and are more likely to receive timely and adequate care should they fall sick. Wealth and risk are distributed unevenly and unfairly by social and technological structures that seem to operate autonomously. These structures are pervasive and deeply intertwined with technologies of bureaucratic control in vivid (as automated decision systems for welfare services^6^ and predictive policing)^7^ and banal forms (as accounting norms, quarterly shareholder dividends, and unscalable unemployment insurance portals running on COBOL).^8^

The risks that define contemporary life are not just socially produced, but are actively managed by technocrats and technologies that calculate risk thresholds for pollutants and automotive safety, by a financial system that creates a market for risk so that it can be hedged against, and by business managers who offer customers the ability to outsource infection risk by arbitraging weak and outdated labor laws. Caitlin Zaloom, an anthropologist who studies risk, identifies two modes of understanding risk from a sociocultural perspective—how groups understand risk as a threat or vulnerability, and how groups attempt to exert control over an uncertain future by limiting exposure to risk.^9^ We apply those understandings of risk to applications of machine learning in the COVID-19 pandemic to ask how those who build machine learning applications construct and act upon risk. This necessarily leads us to also ask how they maintain and build socioeconomic power by exerting control over the distribution of risk. This framing extends ongoing conversations about the social implications of machine learning beyond those of algorithmic bias^10^^,^^11^ to point to a type of harm that occurs not when machine learning “gets it wrong” due to various forms of bias. Rather, we interrogate what happens when machine learning “gets it right” by acting as expected but doing so while reinforcing and exacerbating social and economic inequity.^12^ Despite recent debates within the machine learning community showing how resistant some quarters are to acknowledging the social, cultural, and economic dimensions of the field (against the ample evidence from others),^13^ the question of how to properly bound which aspects of sociotechnical systems machine learning practitioners have agency upon and responsibility for has become vitally important.

Pandemics are not strictly a microbial phenomenon, they follow the contours of society the contagion encounters,^14^ some of which shift medical risk toward the impoverished or oppressed. As each of us are made responsible to minimize our own risk of spreading the coronavirus, it becomes all too clear that “responsibility” is, to some extent, coterminous with a social and economic status that allows us to externalize risk onto others. In recent years, this capacity for nearly autonomous, unquestioned risk externalization has been mediated through machine learning applications to a remarkable extent. Machine learning, because of its versatility in dealing with problems across domains, because of the wealth of financial and computing resources at its practitioners' disposal, and because teams of machine learning engineers can collaborate remotely, has found no shortage of potential applications to the current crisis. Therefore, we suddenly find our personal and societal responses to the pandemic emerging through machine learning applications that act as a ubiquitous tool for distributing societal, health, and economic risks.

Machine learning applications already shape the distribution of risk across the labor, health, and surveillance landscapes that are central to distributing risk as part of our pandemic response. Gig worker platforms, instant delivery, supply chain management, and automated scheduling applications determine who is exposed to how much risk from the disease, while the workers themselves attempt to navigate continually shifting affordances on those platforms.^15^ The machine logics of these systems are determinative of risk, whether human technocrats could have orchestrated these processes in identical ways or not, and early evidence suggests some of the ways that such applications are proving brittle in their response to the COVID-19 pandemic.^16^ These applications financialize and arbitrage risk from app users (whether individual consumers or enterprise users) to gig workers, waged staff, and warehouse laborers, with stockholders reaping the difference.

The tech companies that are driving the technological response to the COVID-19 pandemic have arranged their business models and organizational practices around building products that distribute upsides and downsides according to socio-historical patterns, as interpreted by machines, yet lack the capacity to “understand just how pervasively… technology is being used to marginalize many groups of people,” as leading machine learning researcher Timnit Gebru and colleagues have observed.^13^^,^^17^ Machine learning applications are ultimately exercises in distributing attention and resources, both in how we understand the risks we face and how those risks are distributed across society.

## Risk and Machine Learning

Increasingly, the calculation, production, and management of risk has been accomplished through the application of machine learning techniques. Machine learning is used to understand threats and vulnerabilities, and also as a means of exerting control over such threats. Indeed, risk is foundational to machine learning. Loss functions, central to the applied power of machine learning, were developed to analyze and minimize risk. All objective functions can be thought of as minimizing the risk of a prediction being wrong, but machine learning has been applied to more “human readable” understandings of risk across a vast array of domains. These include predicting cardiovascular risk,^18^ estimation of genetic risk factors,^19^ consumer credit risk,^20^ and risk of individuals attempting suicide.^21^ Machine learning is used not only to understand the nature of threats to health, safety, and finance but also to intervene in these threats by allocating scarce resources toward interventions that minimize risk the most for those who control or own the algorithmic tools. In this way, a rideshare platform produces risk for drivers who take on the liability of car payments and vehicle maintenance, while minimizing risk for the platform itself through its freedom from maintaining a fleet of vehicles for the service it ostensibly offers.^22^

Machine learning, then, is deeply implicated in the social production of risk that Beck and Zaloom describe above. By seeing machine learning as productive of risk, it becomes possible to recognize the responsibilities machine learning practitioners hold for the ways they produce risk. In constructing representations from data, in crafting classifiers and evaluating their utility, and in optimizing for desired performance behaviors, machine learning shapes and distributes risk across society.^23^ Machine learning can identify for bankers which individuals are at the greatest risk of not repaying loans, for police which neighborhoods are at the greatest risk of certain kinds of criminal activity, and for doctors (and insurers) patients who are at the greatest risk of diabetes, heart disease, cancer, pneumonia, or COVID-19. And in all too many cases, machine learning actively *produces* risk while also distributing it unevenly across society, as predictive policing algorithms focus police attention on already over-policed (largely Black and Latinx) neighborhoods.^24^

Algorithmic methods for deciding where risk lies would be a purely academic exercise if not for the real-world impacts to people's lives that this entails. Machine learning applications operate upon theoretical constructs that are not directly observable—creditworthiness, health, and recidivism cannot be directly measured—but rather are inferred from proxies for such constructs.^25^ Models built from such proxies, because they are operationalized as part of algorithmic decision-making systems, often seem like a concrete instantiation of the theoretical constructs they purport to represent but may instead become like self-fulfilling prophecies, actually producing the phenomena they purport to measure. And they are often used in ways that produce concrete negative impacts to people's lives.

Individuals classified as risky loan applicants have trouble buying homes and building wealth. Neighborhoods classified as high-risk crime areas get policed more heavily and see more arrests for petty crimes.^26^ Over time, heavily policed neighborhoods see more re-arrests, and therefore steeper penalties for residents who become re-offenders.^27^^,^^28^ Patients classified as being at high risk of certain diseases may receive life-saving early testing and have better long-term outcomes, but may also see higher healthcare costs and dangerous side effects from maintenance medications,^29^ be seen as having pre-existing conditions by their insurers, or be triaged to a lower degree of medical urgency by a racially biased algorithm trained to seek price efficiency.^30^

To be clear, these impacts are not a first-order result of machine learning but neither are they entirely external to machine learning. Rather, they are the result of complex sociotechnical responses to risk in specific domains. The role of machine learning in producing “risk” as an actionable construct for some and not for others cannot be ignored, as machine learning practices rely on, recreate, and often amplify already-existing patterns of how risk is distributed across society, irrespective of whether that risk is fairly or justly distributed. This is particularly apparent in how racial disparities are algorithmically encoded in many machine learning applications, as when over-representation of non-white inmates in criminal justice records leads to the over-estimation of risk for non-white defendants in pretrial detention models.^31^ However, it is also evident in how such applications themselves participate in the perpetuation of unjust institutions, such as the carceral system.^7^ On top of this, the role that machine learning plays in producing crime, for some and not for others,^32^ cannot be ignored.

Machine learning practitioners have invested significant effort in adjusting technical systems to blunt downstream risks, but the role machine learning might play in the reproduction of patterns of risk is also operative in less-apparent ways as well. Even when datasets are balanced,^33^ optimization functions are constrained to minimize bias for disadvantaged groups,^34^ and instances of algorithmic bias are measured and mitigated,^35^ a long tail^36^ of social effects remain embossed on the data creation and collection processes that underlie the machine learning economy.

## Machine Learning and COVID-19

Given the profound threat of COVID-19, it is crucial to consider how the application of machine learning to the social challenges of a global pandemic can produce and distribute risk across society. These risks are socially constructed, as are specific harms produced by machine learning systems, and so we must ask what is being done to make sure that one is not amplifying the other.

Since the pandemic was declared in early March of 2020,^37^ hundreds of articles have been published to pre-print archives, such as aRxiv, bioRxiv, and medRxiv reporting potential advances in machine learning applications for combatting the pandemic. These applications include natural language processing for combing through the existing literature on COVID-19,^38^ machine learning models that attempt to infer the asymptomatic spread of the virus,^39^ models of the effect of quarantine policies on viral transmission,^40^ facial recognition applications for use in emergency room triage,^41^ and deep learning for COVID-19 diagnoses through medical imaging.^42^ These studies point to the ways machine learning participates in how risk is both understood and managed, through attempts to understand the nature of the threat and to respond to it.

More recently, a host of machine learning applications have been developed to track, treat, and limit the spread of the virus.^43^^,^^44^ These applications include automated contact tracing used to notify those who have been exposed that they ought to self-quarantine (for which machine learning algorithms assist in estimating the strength of contacts between people based on Bluetooth signal strength),^45^^,^^46^ but also natural language processing-based early warning systems (available on a subscription basis) for outbreaks,^47^ and computer vision systems that detect mask-wearing and crowding on public transportation systems to inform potential riders that they may want to choose alternate modes of transportation during busy times.^48^

While these efforts are doubtless well intentioned, each suggests a machine learning intervention into the already-existing distribution of risks and potential harms for society.^49^ In the US we are seeing the most severe cases of COVID-19 striking Black and Latinx communities in drastically disproportionate ways,^50^ and it is clear that this is in large part because of how risk and inequality map onto each other along racial dimensions.^51^ This is in part because of long-standing disparities in health outcomes for Black and Latinx communities, environmental racism that places environmental determinants of health near Black and Latinx communities,^52^ as well as the disproportionate number of Black and Latinx workers in job roles that cannot be filled at home and do not have adequate sick leave or healthecare.^53^ For contact tracing, the ability to make use of a notification to minimize one's own risk by self-quarantining is far too dependent on one's personal wealth and capacity to afford to stay home (either because of a generous workplace sick leave policy, the ability to work from home, or one's own savings). Any contract tracing is a *sociotechnical* system that depends on how different parts of social life fit together—telling people they *should* stay home does not mean that they *will be able to* stay home. For contact tracing to work at all, its designers must be attuned to the context of social life in which such systems can produce harmful, difficult-to-foresee effects^54^ that replicate or amplify inequalities already present in society. Rather than individualizing the risk through contact tracing systems currently being proposed, attending to the contextual use of such a system could collectivize risk by identifying and emphasizing the necessary forms of social support for self-quarantine and medical care: adequate sick leave and quarantine leave policies, robust testing, and economic relief that targets individual workers over large companies.

Automated contact tracing notifications are useful to those who already possess the means to work from home and are less useful to those who cannot self-quarantine without losing their job. An automated update about a crowded subway car will not help someone who has no other means of transportation and cannot be late to return home to care for a child. Others, meanwhile, will maintain their freedom of movement—the delta between who can move about freely and who cannot will have profound consequences for those who have and those who do not have compatible mobile devices, or who lack the resources to maintain their income or care for their dependents while unable to work or cohabitate with others.^55^ In this way, automated technologies that construct and manage the shared risks of the pandemic can perniciously codify and reinforce unjust social and economic dynamics that are the context in which the infectious disease spreads. Machine learning applications may construct risk such that individuals can act upon it to their own advantage without addressing the social conditions that make such risks unevenly distributed, thereby presenting a false sense of risk reduction. Those who build and deploy automated tools to track and treat the pandemic should not treat risk as if it were flat across the population, but as something that they are actively engaged in constructing and distributing and are responsible for doing so justly.

## Managing Risk in the Tech Industry

Understandings of how the production and distribution of risk through algorithmic technologies leads to sociotechnical impacts of machine learning are still in their infancy, and such technologies are only just beginning to be thought of as capable of being brought under any sort of governance regime.^56^, ^57^, ^58^, ^59^ Professional organizations have attempted to mitigate harmful impacts from applying data-driven and machine learning solutions to the problems of the COVID-19 pandemic. Some of these are quite robust, if non-binding, sets of recommendations,^60^ while others attend to a limited definition of privacy rights without attending to the range of sociotechnical impacts discussed above.^61^

Over the past 2 years, we have been studying how those inside of Silicon Valley tech companies, which build the machine learning models that are most likely to directly affect people, go about understanding the impacts of machine learning and developing organizational practices to manage the effect they have on how risk is distributed across society.^62^^,^^63^ Under the mantle of “ethics,” Silicon Valley companies manage the emerging risks their products and services pose for individuals, society, and for their own firms. In response to the unprecedented public health challenges represented by the COVID-19 pandemic, many of these companies are rushing to play a role in producing technological solutions. In the rush to produce solutions, however, it is even more important to think through the lessons the tech industry has learned from managing organizational risk in the years leading up to the current crisis, and not to jettison those lessons out expediency. Reading the potential applications of technology to the current crisis through the recent history of tech ethics, several lessons stand out: context is key, the upside benefits of technology are in tension with downside risks, and leadership and organizational culture matters. These lessons are made explicit through the applications of machine learning to the COVID-19 pandemic, but can also be extended to examining how machine learning, and digital technologies more broadly, produce and distribute risk across society.

### Context Is Key

Any framework to identify and manage risk within an organization must deliberately and methodically consider the context in which it operates. Context includes not just the social milieu at which a product is targeted, but also the intentions, worldview, and the necessarily partial knowledge of those who build the product.^64^ Including producers of technology in any consideration of context is important because the way they are positioned in the world can have an outsized influence on what are chosen as problems to be solved,^65^ what data are selected to serve as proxies for unobservable phenomena,^25^^,^^66^ and what forms of risk are visible. This is particularly true for the use of technology to manage the risks COVID-19 presents to society.

Technological applications for the pandemic offer a compelling set of technical challenges for engineers and designers, but not all technical challenges present opportunities to improve outcomes. A deep learning tool for hypoxia detection intended for use in emergency room triage sounds useful, but given the speed at which health professionals can visually detect hypoxia (by noting pallor of complexion or blue lips),^41^ such a technological intervention is an *additional* step on top of what needs to happen in triage already, not necessarily a *time-saving* tool for busy hospital workers. Recent research demonstrates the importance of recognizing the additional work integrating new tools into the existing practices of workplaces they are intended for requires from all those who interact with them.^67^

Given the enthusiasm for using COVID to accelerate the adoption of artificial intelligence (AI) in healthcare settings,^47^ it is reasonable to ask whether solving relatively simple diagnostic (but scientifically interesting) tasks is really as useful as predicting and managing resources that vulnerable human caretakers truly need, such as adequate personal protective equipment.^68^ Perhaps diagnostic applications of AI receive so much attention because diagnosing is a type of activity that powerful and economically valued physicians do, whereas supply chain management that keeps the nurses, janitors, and technicians—who have far more contact with patients than physicians—safe is mundane and less economically valued. There is less money to be had in keeping nurses alive than in displacing physician labor, for no reason other than how risk has been financialized and distributed. Technologists should ask themselves: if an automated tool for tracking and treating a pandemic is not useful for the most vulnerable, then in what sense is it useful enough to merit investment and justify the effort of integrating it into existing practices?^67^

### Upsides and Downsides

Inside product and legal teams at tech companies, it has often been easier to argue for limiting the risk of a product that might harm users than it is to argue for changes to a product that benefit users. This is particularly true if those positive outcomes for users or society cannot be straightforwardly accounted for in the company's bottom line. This is at least in part because limiting the riskier aspects of a product aligns with mechanisms companies already have to limit their liabilities. Conversely, additional investment in a product “merely” to generate a social good is seen as reducing the return on investment because it raises the cost of investment without raising the financial return in a manner that can be booked. Amid the current pandemic, however, this tendency is inverted, and within the closed loop of technology vendors and enterprise clients, it may appear there is only upside to adapting the ad-serving and data brokerage surveillance apparatus to the purpose of contract tracing or epidemiological modeling. However, this upside comes with significant downside risks that COVID-19 tracking systems might pose to individuals and groups outside that closed loop in the near and distant future.^69^

These risks are often framed as threatening privacy, and there are various technical methods for limiting such risks for individuals, including differential privacy, encrypted computation, and decentralized computation (particularly for contact tracing and other diagnostic applications). Yet such technical methods have trade-offs in terms of accuracy and time-to-market—a not insignificant issue given the urgent demand and short contracting windows for such systems. But “privacy” is an inadequate frame for these risks, because it individualizes responsibility to manage one's own data without attending to how these risks are produced through the design of technical systems and their integration into society. Furthermore, privacy unburdens the legal system from adequately protecting the rights of those who might be harmed by the systematic misuse of personal data, and forestalls any possibility of “collective determination over the infrastructures and institutions that process data and that determine how it will be used.”^70^ Having a framework in place to work through the internal and external risks to the firm, and to society, is crucial for maximizing the upside benefits of any machine learning applications.

To accomplish these aims, such a framework would need to be supported by the necessary resources to achieve the substantive outcomes that are desired. For a company building an intervention as sweeping as contact tracing, success depends on having financial, social, emotional, and medical resources already in place to enable people to deal with the risk a digital contact tracing app assigns to them. A smartphone alert is useless if people do not have the ability to isolate themselves without suffering or failing those who depend on them, which means that the distribution of those capacities also determines the distribution of the upsides and downsides of a contract tracing interventions.^71^ Machine learning applications are (thus far) not useful for rectifying this type of risk distribution despite that there are plenty of data proxies for economic inequity. Because so many machine learning applications are built around arbitraging risk from an advantaged to a disadvantaged party we should expect the same from pandemic solutions that are not subject to critical interrogation.

Therefore, product managers, engineers, policy teams, legal consuls, executive boards, marketing teams, and user experience researchers need to ask themselves if the necessary social support is in place where their product is intended to be used. They also would need to ask themselves if their product will conceive of and distribute risks in a manner that is just, and what measures, metrics, and other signals they would need access to know that such questions have been answered adequately. While some companies have begun to invest in the capacity to ask such questions,^62^^,^^63^ the industry as a whole does not yet have such frameworks in place.

### Leadership Matters

Approaches that can identify and redistribute risk so that it does not fall disproportionately on those least able to bear its burden exist,^72^ but are often not required to be brought into practice. Inside tech firms, choices about investing in upside benefit over limiting downside risk get made at the top, as are decisions about how much time, energy, and cost to invest into context alignment. As our research indicates, without clear signals from the CEO that efforts to enact good governance, build products with responsible safeguards, and prioritize users' rights would not be scrapped for a bottom-line calculation, those efforts cannot amount to much.^62^ In recent years, activist employees and a vigilant public have provided a check^73^ on some companies when their CEOs pursue contracts with repressive regimes or military applications. However, not all companies have experienced this kind of pressure internally (the possible reasons for this run the gamut from a lack of diversity inside to an environment in which such dissent is actively discouraged), and others have not proven responsive when faced with pressure. Some of the most recalcitrant of these companies are in the running for contract tracing application contracts, and will require a different form of pressure to conform to expectations for the responsible deployment of such a system (if this is even possible).

Leadership matters when it comes to the government response to pandemic disease, and when it comes to provisioning contact tracing algorithmic systems from tech companies, too. Many companies, big^74^ and small,^75^ are racing to build contact tracing applications and related infection tracing tools for governments to use. Some will be safer for users, in terms of digital rights, than others. Having options is great, but there is little obligation for federal, state, or local governments to choose the option that is best able to preserve digital rights. As it stands in the US, there are very few legal protections for civil rights and liberties that might be harmed by the misuse of the kind of data contract tracing systems depend on. Draft legislation, like the NY State Geolocation Tracking Ban^76^ would provide a modicum of protection from unreasonable police use of such data, but government contracts for contract tracing systems will be written under the legal frameworks we have now, not the ones we wish we had.

Trust in those at the very top—of companies, of public health institutions, and (perhaps most importantly) national governments—to do what only leadership can do is paramount. Only those who hold final authority can prioritize alternatives that distribute risk equitably rather than toward those who are most vulnerable. In practice this can mean only building in contexts where the necessary social infrastructure for product goals to be met can be ensured. It can also mean refraining from turning the tools of emergency response to COVID-19 toward other, more nefarious purposes. In the absence of regulation, or other forms of social pressure, the incentives for pursuing opportunities to squeeze additional profit by selling data or licensing a machine learning product to unsavory actors can be difficult to resist. Similarly, the resolve needed to dismantle emergency tools when the crisis passes is currently the limiting factor for leadership in determining how the risks we all face of getting sick, losing loved ones, and losing our livelihoods unfolds.

## Conclusion

Historically, risk distribution is a key conceptual and economic feature of machine learning applications. This type of risk is not a natural phenomenon, such as where lightning might strike, but a social construction of technocratic systems through which people must pass to have access to the economy, justice system, and health care. Machine learning has already shaped the landscape on which our society is responding to the COVID-19 pandemic by distributing risk, and the pandemic has accelerated the role that data-driven technology has in directly determining the conditions of our lives. However, despite the efficiency and utility promised by machine learning applications, there lurks a fundamental challenge: are machines good for distributing risk in the ways we actually should distribute it?

Consider for a moment, whether it is possible to build machine learning applications that distribute risk *up* the socioeconomic ladder rather than *down*. Not just “is it conceivable,” but would those of us involved in the research and development of these technologies know what that looks like and how to get there? This would, in part, look like subverting existing power hierarchies, as demonstration projects tracking white-collar crime zones have done.^32^ It would also look like reconfiguring *who* builds machine learning applications (machine learning teams in industry, and corporate ethics teams, have had notorious difficulties retaining Black and Latinx team members)^77^ and how they are governed. If not, then is it just to build high-tech pandemic solutions that distribute risk only downward?

Approaches that can identify and redistribute risk so that it does not fall disproportionately on those least able to bear its burden exist,^72^ but are not required to be brought into practice. Inside tech firms, choices about investing in upside benefit over limiting downside risk get made at the top, as do decisions about how much time, energy, and cost to invest into context alignment. As our research indicates, without clear signals from the CEO that efforts to enact good governance, build products with responsible safeguards, and prioritize users' rights would not be scrapped for a bottom-line calculation, those efforts cannot amount to much.^62^ In recent years, activist employees and a vigilant public have provided a check^73^ on some companies when their CEOs pursue contracts with repressive regimes or military applications. But not all companies have experienced this kind of pressure internally, and others have not proven response when faced with it. Some of the most recalcitrant of these companies are in the running for contracts applying machine learning to the COVID-19 pandemic, and will require a different form of pressure to conform to expectations for the responsible deployment of such a system—if this is even possible.
